# Multiple metastases of androgen indifferent prostate cancer in the urinary tract: two case reports and a literature review

**DOI:** 10.1186/s12920-022-01267-z

**Published:** 2022-05-21

**Authors:** Tsukasa Masuda, Takeo Kosaka, Kohei Nakamura, Hiroshi Hongo, Kazuyuki Yuge, Hiroshi Nishihara, Mototsugu Oya

**Affiliations:** 1 Department of Urology, Keio University School of Medicine, 35 Shinanomachi, Shinjuku-ku, Tokyo, 160-8582 Japan; 2 Genomics Unit, Keio Cancer Center, Keio University School of Medicine, Tokyo, Japan

**Keywords:** Neuroendocrine differentiation prostate cancer, Urinary tract metastasis, *AR*, *TP53*, *BRCA2*, *PTEN*, Aggressive variant prostate cancer

## Abstract

**Background:**

Prostate cancer (PC) is mainly known to metastasize to bone, lung and liver, but isolated metastases of prostate cancer, including ductal carcinoma, in the urinary tract are very rare. We describe two patients with nodular masses in the urinary tract (the anterior urethra or the urinary bladder) that were found on cystoscopy during treatment of castration-resistant prostate cancer.

**Case presentation:**

In both cases, the pathological diagnosis from transurethral tumor resection showed that they were androgen indifferent prostate cancer (AIPC), including aggressive variant prostate cancer (AVPC) in Case 1 and treatment-induced neuroendocrine differentiation prostate cancer (NEPC) in Case 2. In Case 1, Loss of genetic heterozygosity (LOH) of *BRCA2 and* gene amplification of *KRAS* was identified from the urethra polyps. In Case 2, homozygous deletion was observed in *PTEN*, and LOH without mutation was observed in *RB1*.

**Conclusion:**

These are the first reports of two cases of urinary tract metastasis of AIPC.

**Supplementary Information:**

The online version contains supplementary material available at 10.1186/s12920-022-01267-z.

## Background

Prostate cancer (PC) primarily metastasizes to bone, lung, and liver. Reported cases of metastases in the anterior urethra or bladder are rare, including only 15 cases of anterior urethra metastasis. Furthermore, androgen indifferent prostate cancer (AIPC), the pathological characteristics of which have increasingly been described, [1, 2] often metastasizes to similar sites, but there are no reports of urinary tract metastasis. We report two cases involving AIPC metastasis to the urinary tract, describe the genomic sequence, and discuss the potential mechanism of metastasis to the urinary tract.

### Case presentation

**Fig. 1:**
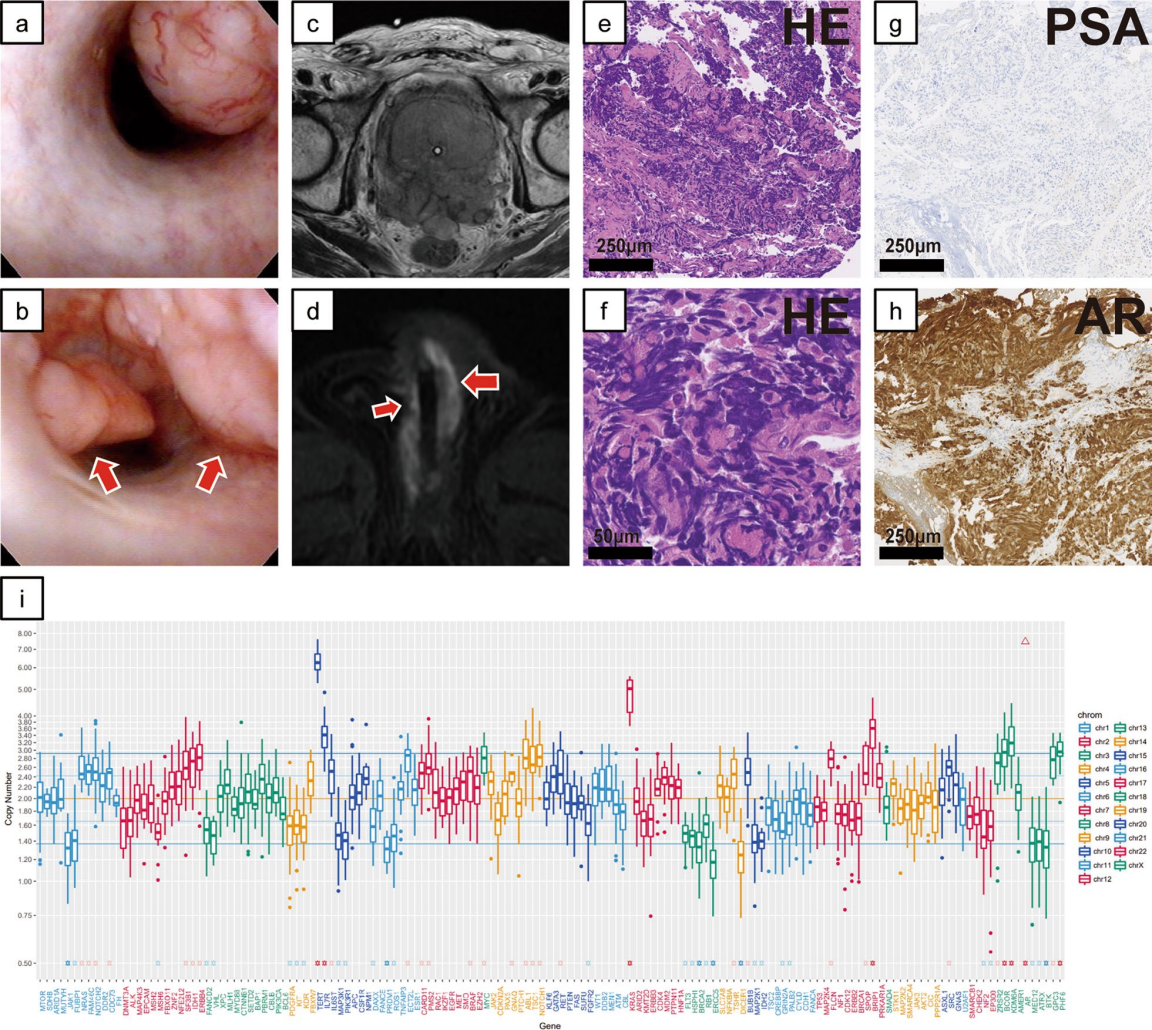
Cystoscopic, imaging, and pathological examination results and genomic sequencing in Case 1. **a**, **b** Cystoscopic findings in the urethra. The cystoscope shows several nodular polyps in the proximal penile urethra and distal bulbar urethra. **c** Prostate magnetic resonance imaging (MRI). The prostate was almost entirely replaced by the tumor, which has invaded the rectum. **d** MR image of the urethra. Metastases of the prostate cancer extended with skip lesions along the corpus spongiosum in the entire anterior urethra. **e**–**h** Representative microscopic images of hematoxylin and eosin (HE) staining and prostate-specific antigen and androgen receptor immunohistochemical staining of transurethral resections of urethra tumor specimens. These images were obtained using the following equipment: microscope, BX53; objective lens, UPLXAPO; camera, DP27; adapter, U-TV1XC. NanoZoomer-XR C12000 was used as acquisition software and the measured resolution was 500 dpi. **i** Examined genes (horizontal axis) and the copy number in Case 1 (vertical axis)
  Targeted next-generation sequencing using an in-house assay of the resected specimen from the urethra polyps was performed (Additional file 1). A *TP53* somatic point mutation (p.H193Y) was detected as a pathogenic variant. Gene amplification was detected in androgen receptor (*AR*) and *KRAS* (estimated copy number (CN): 35.3, 5.8, respectively). Loss of genetic heterozygosity (LOH) without mutation was observed in *BRCA2*. CN variation box (Fig. 1i) indicated a high LOH frequency, which is common in homologous recombination-deficient tumors.
- *Case 2*. A 69 years old man was diagnosed with Gleason score 4 + 5 prostate cancer at another hospital. His serum PSA level was 81.5 ng/mL, and his clinical stage from CT and whole-body bone scans was T3aN1M1 (multiple lung and bone metastases). CAB therapy was started, and his PSA decreased to 0.36 ng/mL. However, after 1 year on androgen deprivation therapy (ADT), resistance to castration developed (PSA: 54.22 ng/mL), so docetaxel was started. For ten cycles of chemotherapy, his PSA decreased to a nadir of 17.2 ng/mL but subsequently increased to 84.0 ng/mL. MRI and CT of the abdomen and pelvis (Fig. 2a and b) showed non-muscle-invasive masses in the neck and urinary bladder posterior wall, although chest CT of the lung metastases showed partial responses (PRs). Cystoscopy showed bladder-neck obstruction by a tumor invading from the prostate gland (Fig. 2c) and revealed another group of nodular masses at the left posterior bladder wall (Fig. 2d). He was referred to our hospital for TURP and transurethral resection of the bladder tumor because of hematuria and urinary obstructive symptoms. Both pathological diagnoses of the bladder neck and posterior wall showed neuroendocrine differentiation prostate cancer (NEPC) (Fig. 2e, f, g, and h). Specifically, high mitotic rate cells were detected using immunolabeling for H&E staining, as illustrated in Fig. 2e, and the signals of synaptophysin were found in over 50% tumor cells (Fig. 2h). From the abovementioned points, we identified Case 2 as treatment-induced NEPC (tNEPC). He underwent two cycles of etoposide and carboplatin. However, the disease progressed, and the anticancer treatment was eventually discontinued.

- *Case 1*. A 79 years-old man presented with obstructive lower urinary tract symptoms at another hospital. His prostate-specific antigen (PSA) level was 15.54 ng/mL. Pathological diagnosis from transrectal needle biopsy was adenocarcinoma with a Gleason Score of 5 + 5. Staging computed tomography (CT) scan showed regional lymph node (LN) metastases. He received combined androgen blockade (CAB) therapy initially, but after a decrease in PSA, his levels eventually increased. He was diagnosed with castration-resistant prostate cancer (CRPC) and began enzalutamide but despite continuous treatment for 6 months, chemotherapy was required. However, although chemotherapy initially lowered his PSA, eventually it increased a lot subsequently. He had difficulty urinating smoothly because of disease progression-related obstruction requiring clean intermittent catheterization. Subsequently, he received abiraterone but it was ineffective. Three years after the original diagnosis, he was referred to our hospital for further treatment of CRPC. He had bloody urine and difficulty with self-catheterization for 5 months after starting. Cystoscopy showed several nodular polyps in the penile urethra (Fig. 1a and b). Magnetic resonance imaging (MRI) demonstrated that the tumor had grown to 8 cm in diameter and invaded the rectum (Fig. 1c). MRI also showed metastases of the prostate cancer extended with skip lesions along the corpus spongiosum in the entire anterior urethra (Fig. 1d). Moreover, he developed a catheter obstruction caused by hematuria, so a suprapubic cystostomy tube was placed and transurethral resection of the prostate (TURP) was performed to achieve tumor bleeding coagulation. Simultaneously, he underwent endoscopic resection of the urethra polyps. Histology showed metastasis of prostatic adenocarcinoma with aggressive variant (Fig. 1e, f, g, and h); particularly, as illustrated in Fig. 1e and f with immunolabeling for hematoxylin and eosin staining, the pathological findings of Case 1 exhibited a high-grade tumor defined by characteristic nuclear features, including lack of prominent nucleoli and high nuclear to cytoplasmic ratio. He was treated using cisplatin and etoposide. After two cycles, he achieved a progressive disease and he was treated with the best supportive care.

**Fig. 2:**
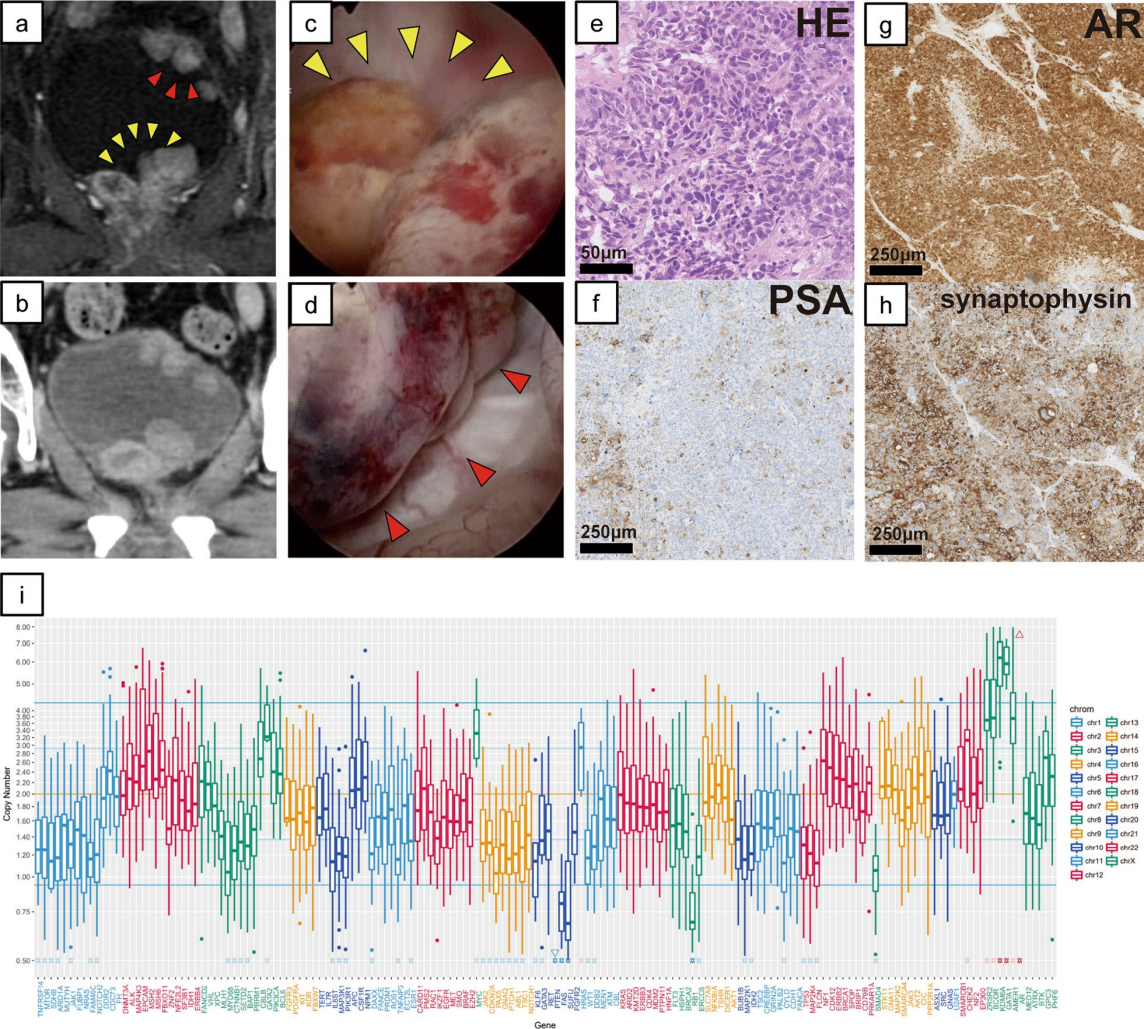
Cystoscopic, imaging, and pathological examination results and genomic sequencing in Case 2. **a** Bladder magnetic resonance imaging. **b** Pelvic computed tomography. Non-muscle-invasive masses in the neck and posterior wall of the urinary bladder are shown. **c**, **d** Cystoscopic findings. The tumor invaded from the prostate gland and has obstructed the bladder neck (**c**) and nodular masses are shown at the left posterior bladder wall (**d**). **e**–**h** Representative microscopic images of hematoxylin and eosin (HE) staining and prostate-specific antigen, androgen receptor, and synaptophysin immunohistochemical staining and transurethral resection of the bladder tumor specimens. These images were obtained using the following equipment: microscope, BX53; objective lens, UPLXAPO; camera, DP27; adapter, U-TV1XC. NanoZoomer-XR C12000 was used as acquisition software and the measured resolution was 500 dpi. **i** Examined genes (horizontal axis) and the copy number in Case 2 (vertical axis)

Targeted next-generation sequencing of the resected specimen from the posterior bladder posterior wall identified a *TP53* somatic point mutation (p.R196P) as a pathogenic variant. Gene amplification was detected in *AR* (estimated CN: 25.4). Homozygous deletion was observed in *PTEN*, and LOH without mutation was observed in *RB1*. The CN variation box is shown in Fig. 2i.

## Discussion and conclusion

Our two patients were initially diagnosed with prostate adenocarcinoma, which during hormonal treatment progressed with aggressive variant and neuroendocrine differentiation and multiple metastases to the urinary tract. NEPC occurs in 17% of patients with metastatic CRPC and has a poorer prognosis than other PCs [1, 3, 4]. NEPC tends to metastasize to bone, lung, and liver, and urethra or bladder metastasis has not been reported [1, 2]. Isolated metastasis or recurrence of PC in the intra-urinary tract is extremely rare, with only 15 cases reported previously [5–19] (Table 1); five had an origin in the prostatic ductal adenocarcinoma (PDC) and most of the rest were from adenocarcinomas with lower Gleason scores. On the other hand, in a study of 282 patients with secondary bladder neoplasms, 19% had PC-related secondary urinary bladder tumors [20], and 39% had urinary bladder metastases at autopsy [21], but the majority showed bladder-neck invasion. Case 2 may be the first report of adenocarcinoma with tNEPC as there are few case reports on isolated metastasis of PC to the bladder except for the bladder neck [22, 23].

**Table 1:** Systematic review of studies of urethral metastasis from prostate cancer cases

No. ref	Case	Age	Symptoms at the time of reccurence	PSA (ng/ml)	Location	Appearance (shape, number, size)	Treatment	Pathology	Possible causes
	*Our case (Case 1)*	*79*	*Hematuria with difficulty urinating*	*841*	*In the proximal penile urethra and distal bulbar urethra*	*Nodular, multiple*	*TUR*	*AVPC*	*CIC*
[5]	Britt Haller et al. [5]	67	Painless hematuria	1.5	Distal bulbar urethra and distal penile urethra in the navicular fossa	Papillary, several	Urethrectomy	PDC	Post TURP/EBRT (4 years)
[6]	Darren J. Bryk et al. [6]	83	Obstructive voiding symtoms	0.67	From the penile to the membranous urethra	Papillary, multiple	Biopsies	PC	Post brachytherapy (9 years)
[7]	Yong G Wang et al. [7]	66	Painless hematuria	0.13	In the anterior bulbar urethra	Papillaey, single	TUR	PDC	Post radiation (4 years)
[8]	Hansan Jhaveri et al. [8]	82	Hematuria and urethral bleeding with difficulty urinating	0.26	From the prostatic urethra past the membranous urethra	A large mass, single	No invasive treatments	PC	Direct extension
[9]	Ibrahim Zardawi et al. [9]	84	Urinary retention and symptoms of urinary tract infection	10.3	One of the lesions in the memvranous urethra, two in the bulbar and penile urethra	Polyp, three lesions	TUR	PC	Post TURP (3 years)
[11]	Darren Beiko et al. [11]	68	Gross hematuria	0.7	In the midbulbous urethra	Polyp, single, 2 mm	Cold cup biopsy	PC	Post TURP/EBRT (4 years)
[10]	Enrique Gomez et al. [10]	68	LUTS and urethral bleeding	1.7	Between the distal bulbar urethra and proximal penile urethra	Nodular, single	TUR	PC	Post radiation (4 years)
[12]	Chi-Feng Hung et al. [12]	77	Voiding straining and a bifurcated voiding stream	5.02	8 cm from the meatus and 2 cm distal to the bulbous urethra	Nodular, single	TUR	PC	Venous spread
[13]	Jutin M. Green et al. [13]	74	Painless hematuria	1.25	In the entire anterior urethra, including the fossa navicularis	Papillary, multiple	TUR	PDC	Post radiation (5 years)
[14]	G. Nabi et al. [14]	65	Gross hematuria	12	In anterior urethra leading to stricture	Multiple nodules with ulcerations	TUR	PC	Post TURP (2 weeks)
[15]	C. Ohyama et al. [15]	71	Gross hematuria	5.2	On the distal urethra	Papillary, small	Chemotherapy TUR	PDC	Unidentified
[16]	T. Kobayashi et al. [16]	76	Gross hematuria	Normal	On the anterior urethra	Nonpapillary, sessile	TUR	PC	Post TURP
[17]	Graeme B Taylor et al. [17]	68	Gross painless hematuria	0.8	In the anterior penile urethra 4 cmfrom the external meatus	Papillary, single	TUR	PDC	Post TURP (3 years)
[18]	Faruk Aydin et al. [18]	84	A watery, bloody urethral discharge	–	The prostatic and anterior penile urethra	Papillary, single	TUR	PC	post TURP (2 years)
[19]	Narendra Kotecha et al. [19]	64	Intermittent spotting of blood	–	In the pendulous urethra, approximately 2.5 cm proximal to the urethral meatus	Papillary, single	TUR	PC	post TURP (2 years)

Italics to distinguish between the past cases and our case

*PC* Prostate cancer, *PDC* Prostate ductal carcinoma, *AVPC* Aggressive variant prostate cancer, *CIC* Clean intermittent catheterization, *TUR* Transurethral resection

From a genomic perspective, we wondered why these two cases progressed so quickly. Some patients with AIPC, including AVPC and tNEPC, respond to platinum-based combination chemotherapeutic regimens, but our patients were relatively treatment resistant. We actually performed targeted genomic sequencing of the formalin-fixed paraffin-embedded tumor specimens from TURP by applying algorithms previously reported [24, 25] and identified the factors common between the two patients: *AR* amplification and *TP53* mutation (Figs. 1 and 2i). *AR* is overexpressed in most CRPC patients, and *AR* amplification means that these patients acquired castration resistance during cancer progression [26]. However, the fact was reported that in AIPC definition that the presence of AR amplification was irrelevant [2, 3]. In particular, our Case 1 patient showed *KRAS* amplification. Although *KRAS* mutation may be an advanced prostate cancer biomarker [27],the importance of *KRAS* amplification is uncertain. Progression of metastatic prostate cancer previously was coupled with enhanced expression levels of enhancer of zeste homolog, which is synergized by activation of *KRAS* and *AR* overexpression [28]. In Case 1, KRAS amplification may have been associated with accelerated de-differentiation to intractable NEPC. In Case 2, *RB1* loss co-occurred with *TP53* mutation. Previous studies have described that *TP53* mutation cooperated with *RB1* loss to confer an ADT-resistant phenotype, proposed as an aggressive variant prostate cancer [29, 30].

There are several hypotheses regarding the mechanisms of urethra metastasis, as in Case 1, including implantation following instrumentation or catheterization [11, 13]. Table 1 shows that previous patients with urethra metastasis had a history of post-TURP or prior radiotherapy for PC (or PDC), but our patient 1 did not respond. Additionally, because the tumor obstructed his urinary tract, he could not urinate smoothly with the self-catheter. Consequently, the mechanism for anterior urethra metastasis in Case 1 could have been direct surface implantation by self-catheterization. Regarding Case 2, it is probable that the mechanism of bladder metastasis was initial NEPC invasion of the bladder neck and subsequent posterior wall seeding. Metastasis from a urothelial carcinoma is well known, but the mechanism is unclear [31]. One hypothesis involves the seeding or intraepithelial spread of transformed cells [31, 32]. The spread from the primary tumor to the bladder wall in Case 2 may be similar to the spread of a urothelial carcinoma. These two cases also involved very interesting metastatic mechanisms.

## Supplementary Information

**Additional file 1**. How to perform the targeted next-generation sequencing using an in-house assay of the resected specimen.

## Data Availability

The data of targeted next-generation sequencing in two cases have been deposited to the Genome Sequence Archive (GSA). The assigned accession number is HRA002209. (https://bigd.big.ac.cn/gsa-human/browse/HRA002209).

## Abbreviations

PC: Prostate cancer
AIPC: Androgen indifferent prostate cancer
AVPC: Aggressive variant prostate cancer
NEPC: Neuroendocrine differentiation prostate cancer
PSA: Prostate-specific antigen
CAB: Combined androgen blockade
CRPC: Castration-resistant prostate cancer
c-TURP: A channel transurethral resection of the prostate
AR: Androgen receptor
LOH: Loss of genetic heterozygosity
ADT: Androgen deprivation therapy
PDC: Prostatic ductal adenocarcinoma
