# Supplementary material for: Multiple metastases of androgen indifferent prostate cancer in the urinary tract: two case reports and a literature review
Source: BMC Med Genomics. 2022 May 21;15:118. doi: 10.1186/s12920-022-01267-z (PMC9124419; doi:10.1186/s12920-022-01267-z)
Supplement: Supplementary file 1 — Additional file 1. How to perform the targeted next-generation sequencing using an in-house assay of the resected specimen. [file 12920_2022_1267_MOESM1_ESM.docx]

**Additional file 1**

We investigated the occurrence of mutations in 160 cancer-related genes in the urological cancers. Sections (10 µm) were dissected to provide > 20% tumor cells in the specimens and to minimize the presence of necrosis. Genomic testing was performed on a PleSSision-Rapid internal clinical sequencing apparatus, which is used for all genome sequencing-related analyses in our hospital (Keio University Hospital). This apparatus was used to extract genomic DNA from tumor samples and peripheral blood mononuclear cells extracted from cancer patients, following the provision of consent to receive comprehensive genomic testing. This study was conducted in accordance with the Declaration of Helsinki and Title 45, U.S. Code of Federal Regulations, Part 46, Protection of Human Subjects, effective December 13, 2001.

DNA quality was checked by calculating the DNA integrity number (DIN) using an Agilent 2000 TapeStation (Agilent Technologies, Waldbronn, Germany) prior to conducting targeted amplicon exome sequencing of the 160 genes implicated in cancer using the Illumina MiSeq sequencing platform (Illumina, San Diego, CA). Sequencing data were entered into the GenomeJack bioinformatics pipeline (Mitsubishi Space Software, Tokyo, Japan) for analysis. Cancer-specific changes in somatic genes, including SNVs, insertions/deletions, and copy number variations were detected and used to determine the TMB.
